# Exosomes for the Management of Low Back Pain: A Review of Current Clinical Evidence

**DOI:** 10.7759/cureus.57539

**Published:** 2024-04-03

**Authors:** Ashim Gupta

**Affiliations:** 1 Regenerative Medicine, Future Biologics, Lawrenceville, USA; 2 Regenerative Medicine, BioIntegrate, Lawrenceville, USA; 3 Orthopaedics, South Texas Orthopaedic Research Institute, Laredo, USA; 4 Regenerative Medicine and Orthopaedics, Regenerative Orthopaedics, Noida, IND

**Keywords:** secretome, mesenchymal stem cells, exosomes, extracellular vesicles, regenerative medicine, facet joint, disc herniation, intervertebral disc, back pain, chronic low back pain

## Abstract

Low back pain affects millions of people, creating an enormous financial burden on the global healthcare system. Traditional treatment modalities are short-lived and have shortcomings. Recently, orthobiologics, including extracellular vesicles or exosomes derived from mesenchymal stem cells, have markedly increased for managing musculoskeletal conditions. Here, the primary aim is to review the outcomes of clinical studies using extracellular vesicles or exosomes for treating low back pain. Numerous databases (Scopus, PubMed, Web of Science, Embase, and Google Scholar) were searched using terms for the intervention ‘exosomes’ and the treatment ‘low back pain’ for studies published in English to March 18, 2024. Articles utilizing exosomes for the management of low back pain were included. Articles not utilizing exosomes, not explicitly stating the presence of exosomes in their formulation, or not targeting low back pain were excluded. Two articles that met our pre-defined criteria were included in this review. The results showed that administering extracellular vesicles or exosomes is safe and potentially effective in patients suffering from low back pain. Yet, more sufficiently powered, multi-center, prospective, randomized, and non-randomized trials with longer follow-up are essential to assess the long-term safety and efficacy of extracellular vesicles or exosomes derived from various sources and to support its routine clinical use for managing low back pain.

## Introduction and background

Musculoskeletal (MSK) injuries are the most common cause of disability, significantly affecting the activities of daily living and overall quality of life [1,2]. Low back pain is one of the leading MSK complaints, impacting millions of individuals worldwide (619 million individuals were affected by 2020, and the number is expected to increase to 843 million by 2050) with a lifetime prevalence of 65-85%, resulting in considerable economic burden (>USD50 billion/year) [3-8]. Back pain occurs for several reasons, with lumbar facet joints identified as a major cause of continuing low back pain, accounting for 15-45% of all chronic low back pain cases [9]. The facet/zygapophyseal/spinal joint encompasses a synovial capsule, synovial membrane, hyaline cartilage, and subchondral bone, which is accountable for tension/compression resistance and mobility [10]. Facet joints degenerate over time due to the secretion of inflammatory cytokines caused by age-related natural intervertebral disc degeneration, irregular body mechanics, and strains or trauma, leading to inflammation and pain and resulting in facet joint syndrome or spondylosis [6,11,12]. Disc herniation, defined as the bulging of the disc into the spinal canal, is another common cause of low back pain and results in compression of the nerve roots, leading to lumbar radiculopathy [13,14]. Existing treatment modalities for managing low back pain include non-pharmacological conservative modalities, such as physical therapy, acupuncture, and chiropractic care; pharmacological agents, such as oral narcotics and non-steroidal anti-inflammatory drugs, and injections for medial nerve blocks consisting of local anesthetics and steroids; and minimally invasive procedures, such as radiofrequency ablation [2,6,15,16]. However, these modalities above have shortcomings, including limited long-term amelioration of symptoms and side effects [2,6,17].

The last decade has seen a marked increase in the use of orthobiologics for regenerative medicine applications, including managing chronic MSK pain conditions [18]. Orthobiologics currently utilized in the clinical practice include autologous peripheral blood-derived orthobiologics, such as leukocyte-rich or poor platelet-rich plasma; adipose tissue-derived formulations, such as stromal vascular fraction; bone marrow aspirate concentrate; and perinatal tissue-derived biologics such as amniotic tissue (membrane and/or fluid), umbilical cord and Wharton’s jelly [2,18-24]. The effectiveness of these biologics is credited to the presence of stem cells and/or bioactive molecules such as growth factors, cytokines, and extracellular vesicles or exosomes [25]. Only a few studies have evaluated the safety and efficacy of extracellular vesicles or exosomes for managing low back pain. This review aims to summarize the outcomes of clinical studies involving the use of extracellular vesicles or exosomes to manage low back pain. The secondary aim is to record the ongoing clinical studies registered on different clinical trial protocol repositories involving extracellular vesicles or exosomes for low back pain treatment.

## Review

Methodology

Search Criteria

**Figure 1:**
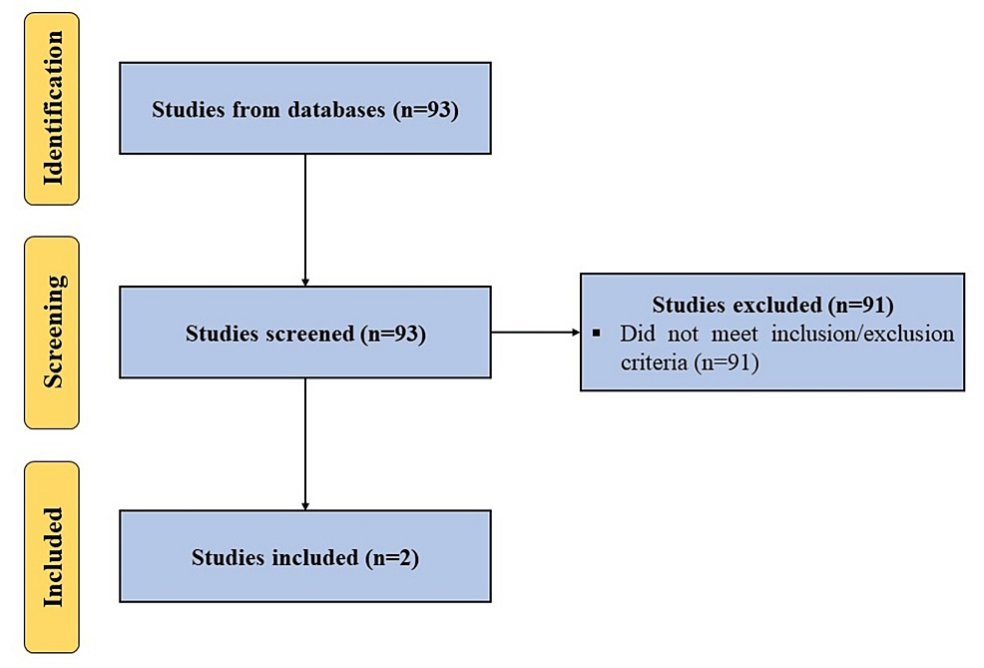
A PRISMA flow diagram outlining the record identification and selection process.

A systematic search, following the Preferred Reporting Items for Systematic Reviews and Meta-Analysis (PRISMA) guidelines, using the terms (‘exosomes’ OR ‘exosome’ OR ‘extracellular Vesicles’ OR ‘secretomes’) AND (‘low back’ OR ‘low back pain’ OR ‘back pain’ OR ‘intervertebral disc’ OR ‘lumbar’ OR ‘disc herniation’), in several databases (Scopus, PubMed, Web of Science, Embase, and Google Scholar) for articles published up to March 18, 2024, in English language, was carried out. All clinical studies utilizing extracellular vesicles or exosomes to manage low back pain were included. Articles not utilizing extracellular vesicles or exosomes OR not explicitly stating the presence of extracellular vesicles or exosomes in their formulation OR not targeting low back pain were excluded. A flow diagram is shown in (Figure 1).

Moreover, we searched different clinical trial protocol registries, including ClinicalTrials.gov, Clinical Trials Registry-India (CTRI), and Chinese Clinical Trial Register (ChiCTR), using the search above terms to find registered ongoing clinical studies involving the use of extracellular vesicles or exosomes for low back pain treatment.

Results

Clinical Studies

Phillips et al. [26], in a single center, open-label, prospective, non-randomized study, evaluated the safety and efficacy of an interlaminar epidural injection of bone marrow mesenchymal stem cells-derived exosomes (BM-MSC-Exo) (10-80 billion extracellular vesicles/mL) in patients with lumbar and cervical radiculopathy, caused by intervertebral disc herniation. 10 participants, five with lumbar and five with cervical radiculopathy, aged 18 years or above, with symptoms of radicular pain for years and confirmation of mild-to-moderate herniated nucleus pulposus with neural foraminal compression via magnetic resonance imaging (MRI), were enrolled. Participants with a history of side effects to epidural steroid injections, stenosis, cauda equina, multifactorial diagnoses of radiculopathy, myelomalacia, or malignancy were excluded. Outcome measures included adverse events, Upper Extremity Functional Scale (UEFS), Brief Pain Inventory (BPI), Lower Extremity Functional Scale (LEFS), Oswestry Disability Index (ODI), and QuickDash (QD) scores, evaluated at baseline and 24-hour, 3 days, 1 week, 3 weeks and 1 month follow up post-injection. Participants reported mild adverse events such as pain and soreness at the injection site and headache, which were resolved within five days post-injection. No severe adverse effects were reported. An average improvement of 55% in BPI, 55.2% in QD, 26% in LEFS, 25.4% in UEFS, and 19.75% in ODI was reported at 1-month follow-up compared to the baseline. This study had limitations, including a small cohort size, short follow-up, lack of statistical analysis, and absence of a control group. Despite these, the results from this study showed that epidural administration of BM-MSC-Exo is safe and potentially efficacious in patients suffering from lumbar or cervical radiculopathy.

Wilson et al. [27], in a single center, open-label, investigator-initiated, prospective, non-randomized study, evaluated the safety and efficacy of single injection in the facet joint space of human bone marrow mesenchymal stem cells-derived extracellular vesicles (BM-MSC-EV) (60-80 billion EVs/mL) in patients with lumbar facet joint pain. Twenty patients with lumbar facet joint pain (L4-L5 or L5-S1) confirmed via review of physical examination, medical history, and MRI imaging were included. Participants with additional cephalad or arthritis at other proximal levels were excluded. Outcome measures included BPI (both severity and interference index) and ODI, assessed at baseline and 24 hours, 3 days, 1 week, 3 weeks, 1 month, 2 months, and 3 months follow-up after injection. No adverse effects were observed. Statistically significant (p<0.0001) improvements in all three outcome measures, BPI-SI (severity index) (65.04%), BPI-II (interference index) (72.09%), and ODI (58.43%), were reported at three months follow-up compared to the baseline. This study also had limitations, including a small cohort size, short follow-up, and absence of a control group. Despite these, the results from this study demonstrated that the administration of BM-MSC-EV is safe and potentially effective in patients suffering from lumbar facet joint pain. The results from the aforementioned studies are summarized (Table 1).

**Table 1:** Summary of main findings of included clinical studies

Author [Reference]	Main Findings
Phillips et al. [26]	The epidural administration of bone marrow mesenchymal stem cells-derived exosomes in patients with lumbar and cervical radiculopathy is potentially safe and led to improvements in the Brief Pain Inventory, Upper Extremity Functional Scale, Oswestry Disability Index, Lower Extremity Functional Scale and QuickDash scores at 1 month follow-up compared to the baseline.
Wilson et al. [27]	The administration of bone marrow mesenchymal stem cells-derived extracellular vesicles in the facet joint space in patients with lumbar facet joint pain was safe and led to significant improvements in the Brief Pain Inventory – Severity Index, Brief Pain Inventory – Interference Index and Oswestry Disability Index scores at 3 months follow-up compared to the baseline.

Ongoing Clinical Studies

As of March 18, 2024, only one clinical trial evaluating the safety and/or efficacy of exosomes for managing low back pain is listed on ClinicalTrials.gov (Table 2). No clinical studies were registered on CTRI or ChiCTR.

**Table 2:** Clinical trials registered on ClinicalTrials.gov until March 18, 2024, evaluate the safety and/or efficacy of exosomes for managing low back pain

Study Identifier	Biologic	Study Phase; Estimated Enrollment (N)	Primary Outcome Measure(s)	Recruitment Status	Study Location(s)
NCT04849429	Platelet-rich Plasma with Exosomes	Phase I; N=30	Visual analog scale (VAS) [*Time Frame: Change from Baseline VAS at 1,3,6,12 months*]	Completed	India
			Roland Morris Disability Questionnaire (RDQ) [*Time Frame: Change from Baseline RDQ at 1,3,6,12 months*]		
			SF 36 health questionnaire [*Time Frame: Change from Baseline SF 36 at 1,3,6,12 months*]		
			Functional rating index [*Time Frame: Change from Baseline Functional rating index at 1,3,6,12 months*]		

Discussion

The absence of efficient gold-standard therapy for managing low back pain poses a significant challenge for clinicians and results in significant disability in patients and a substantial burden on healthcare systems throughout the world [2]. Intra-articular administration of steroids is the most widely used option, but no short-term or long-term benefits, even compared to the placebo, were reported [2]. Lately, cellular therapy involving the use of mesenchymal stem cells (MSCs), derived from various autologous and allogenic sources, including bone marrow, adipose tissue, and perinatal tissue, have shown potential for regenerative medicine applications, attributed to their ability for self-proliferation and multipotency [28]. EVs, including Exos, secreted by MSCs in a paracrine manner, are the key mediators responsible for the therapeutic efficacy of MSCs [29]. EVs or Exos can help overcome certain challenges MSCs present, including insufficient retention and survival at the administration site; unfavorable survival and proliferation in inflammatory and ischemic microenvironment; and release of inflammatory cytokines by dead cells, which may lead to adverse effects on the peripheral cells. EVs or Exos are also known to exhibit anti-inflammatory properties, enhance macrophage polarization to the M2 phenotype, and offer a basis for tissue healing [30]. In addition, EVs or Exos have lower immunogenicity than the MSCs and will not result in immune rejection [31].

The current study reviewed the therapeutic ability of EVs or Exos for managing low back pain. Clinical studies using EVs or Exos for low back pain treatment were included. Based on our pre-defined search terms and inclusion/exclusion criteria, two studies fit the scope of our review (Table 1). Phillips et al. demonstrated that administration (epidural) of BM-MSC-Exo in lumbar and cervical radiculopathy patients is safe and resulted in decreased pain and improved function at 1-month follow-up compared to the baseline [26]. Wilson et al. showed that administration (in the facet joint space) of BM-MSC-EV in lumbar facet joint patients is safe and led to significant improvements in pain and function at three months follow-up compared to the baseline [27]. The results from these studies are in accordance with the literature demonstrating the efficacy of MSCs in ameliorating low back pain [32-34]. Thus, EVs or Exos may have the potential to circumvent limitations posed by MSCs while retaining their regenerative ability.

Only one clinical study was registered on the clinical trial protocol repositories (Table 2). This trial assessed the safety and efficacy of intra-discal injection of Exos derived from the peripheral blood in patients suffering from low back pain. Despite the status of this trial being listed as ‘completed’, to date, no data has been posted on ClinicalTrials.gov or published in a peer-reviewed journal.

## Conclusions

In conclusion, the above-mentioned prospective studies demonstrated that the administration of extracellular vesicles, or exosomes, in the epidural or facet joint space is safe and potentially efficacious in low back pain patients. However, more sufficiently powered, multi-center, prospective, randomized, and non-randomized trials with longer follow-up are essential to assess the long-term safety and efficacy of extracellular vesicles or exosomes derived from various sources and to support its routine clinical use in patients suffering from low back pain.

## References

[1] Storheim K, Zwart JA (2014). Musculoskeletal disorders and the Global Burden of Disease study. Ann Rheum Dis.

[2] Gupta A, Maffulli N (2023). Amniotic membrane and/or umbilical cord tissue for treatment of facet joint syndrome: A narrative review. J Orthop Surg Res.

[3] The Lancet Rheumatology (2023). The global epidemic of low back pain. Lancet Rheumatol.

[4] Fatoye F, Gebrye T, Mbada CE, Useh U (2023). Clinical and economic burden of low back pain in low- and middle-income countries: A systematic review. BMJ Open.

[5] Fatoye F, Gebrye T, Ryan CG, Useh U, Mbada C (2023). Global and regional estimates of clinical and economic burden of low back pain in high-income countries: A systematic review and meta-analysis. Front Public Health.

[6] Norwood SM, Han D, Gupta A (2024). H-wave(®) device stimulation for chronic low back pain: a patient-reported outcome measures (PROMs) study. Pain Ther.

[7] Thiese MS, Hegmann KT, Wood EM (2014). Prevalence of low back pain by anatomic location and intensity in an occupational population. BMC Musculoskelet Disord.

[8] El Melhat AM, Youssef AS, Zebdawi MR, Hafez MA, Khalil LH, Harrison DE (2024). Non-surgical approaches to the management of lumbar disc herniation associated with radiculopathy: A narrative review. J Clin Med.

[9] Perolat R, Kastler A, Nicot B (2018). Facet joint syndrome: From diagnosis to interventional management. Insights Imaging.

[10] Varlotta GP, Lefkowitz TR, Schweitzer M, Errico TJ, Spivak J, Bendo JA, Rybak L (2011). The lumbar facet joint: A review of current knowledge: Part 1: Anatomy, biomechanics, and grading. Skeletal Radiol.

[11] Alexander CE, Cascio MA, Varacallo M (2024). Lumbosacral Facet Syndrome. https://pubmed.ncbi.nlm.nih.gov/28722935/.

[12] Igarashi A, Kikuchi S, Konno S, Olmarker K (2004). Inflammatory cytokines released from the facet joint tissue in degenerative lumbar spinal disorders. Spine (Phila Pa 1976).

[13] Medina-Pérez JJ, Vega-Rosas A, Coubert-Pelayo SG, Rosas-Barcelo LS (2023). Cooled radiofrequency treatment for radicular pain related to lumbar disc herniation. Cureus.

[14] Dydyk AM, Khan MZ, Singh P (2022). Radicular Back Pain. https://www.ncbi.nlm.nih.gov/books/NBK546593/.

[15] Manchikanti L, Boswell MV, Singh V, Pampati V, Damron KS, Beyer CD (2004). Prevalence of facet joint pain in chronic spinal pain of cervical, thoracic, and lumbar regions. BMC Musculoskelet Disord.

[16] Morlion B (2013). Chronic low back pain: Pharmacological, interventional and surgical strategies. Nat Rev Neurol.

[17] Cooke M, Tan EK, Mandrycky C, He H, O'Connell J, Tseng SCG (2014). Comparison of cryopreserved amniotic membrane and umbilical cord tissue with dehydrated amniotic membrane/chorion tissue. J Wound Care.

[18] Gupta A, Migliorini F, Maffulli N (2024). Management of rotator cuff injuries using allogenic platelet-rich plasma. J Orthop Surg Res.

[19] Gupta A, Jain V (2024). Autologous conditioned plasma (ACP) and osteoarthritis of the knee: A review of current clinical evidence. Cureus.

[20] Gupta A, Aratikatla A (2024). Hyperacute serum and knee osteoarthritis. Cureus.

[21] Gupta A (2024). Autologous protein solution (APS) and osteoarthritis of the knee: A scoping review of current clinical evidence. Cureus.

[22] Muthu S, Jeyaraman M, Narula A (2023). Factors influencing the yield of progenitor cells in bone marrow aspiration concentrate-A retrospective analysis of 58 patients. Biomedicines.

[23] Weninger P, Feichtinger X, Steffel C, Kerschbaumer C, Duscher D (2023). Arthroscopy with lipoaspirate and plasma infiltration using adipose-derived stem cells plus platelet-rich plasma: Harvesting and injection for arthroscopic treatment of cartilage defects of the knee. Arthrosc Tech.

[24] Aratikatla A, Maffulli N, Rodriguez HC, Gupta M, Potty AG, El-Amin SF 3rd, Gupta A (2022). Allogenic perinatal tissue for musculoskeletal regenerative medicine applications: A systematic review protocol. J Orthop Surg Res.

[25] Gupta A (2022). Commentary: Cell-free stem cell-derived extract formulation for treatment of knee osteoarthritis. J Orthopedics & Orthopedic Surg.

[26] Phillips A, Wong A, Chen G, LaSalle J, Kuo J (2021). One month safety study of ExoFlo injection for the treatment of lumbar or cervical radiculopathy in the epidural space. IJSRA.

[27] Wilson JE, Today BA, Salazar M (2024). Safety of bone marrow derived mesenchymal stem cell extracellular vesicle injection for lumbar facet joint pain. Regen Med.

[28] Xia Y, Yang R, Hou Y, Wang H, Li Y, Zhu J, Fu C (2022). Application of mesenchymal stem cell-derived exosomes from different sources in intervertebral disc degeneration. Front Bioeng Biotechnol.

[29] Witwer KW, Van Balkom BW, Bruno S (2019). Defining mesenchymal stromal cell (MSC)-derived small extracellular vesicles for therapeutic applications. J Extracell Vesicles.

[30] Arabpour M, Saghazadeh A, Rezaei N (2021). Anti-inflammatory and M2 macrophage polarization-promoting effect of mesenchymal stem cell-derived exosomes. Int Immunopharmacol.

[31] Elahi FM, Farwell DG, Nolta JA, Anderson JD (2020). Preclinical translation of exosomes derived from mesenchymal stem/stromal cells. Stem Cells.

[32] Miranda L, Quaranta M, Oliva F, Maffulli N (2023). Stem cells and discogenic back pain. Br Med Bull.

[33] Schneider BJ, Hunt C, Conger A (2022). The effectiveness of intradiscal biologic treatments for discogenic low back pain: A systematic review. Spine J.

[34] Xie B, Chen S, Xu Y (2021). Clinical efficacy and safety of human mesenchymal stem cell therapy for degenerative disc disease: A systematic review and meta-analysis of randomized controlled trials. Stem Cells Int.

